# Factors Defining the Association Between Vitamin D and Testosterone in Males With Type 2 Diabetes and Hypogonadism

**DOI:** 10.3389/fendo.2022.842722

**Published:** 2022-04-12

**Authors:** Xin Hu, Xue Han, Yu Chen, Pingping Xiang, Xiao Wei, Tong Gong, Zhiwei He, Yafeng Su, Guofang Chen, Chao Liu

**Affiliations:** ^1^ Endocrine and Diabetes Center, Affiliated Hospital of Integrated Traditional Chinese and Western Medicine, Jiangsu Province Academy of Traditional Chinese Medicine, Nanjing University of Chinese Medicine, Nanjing, China; ^2^ Key Laboratory of Tradicional Chinese Medicine Syndrome and Treatment of Yingbing of State Administration of Traditional Chinese Medicine, Jiangsu Province Academy of Traditional Chinese Medicine, Nanjing, China

**Keywords:** vitamin D, testosterone, males, type 2 diabetes, hypogonadism

## Abstract

**Objective:**

This study aimed to evaluate Serum 25-hydroxyvitamin D (25[OH]D) levels in diabetic men with and without hypogonadism and figured out the potential factors influencing the connection between vitamin D and testosterone.

**Methods:**

A total of 367 men with type 2 diabetes mellitus (T2DM) were investigated, including 254 men with normal gonadal function (Group 1) and 113 men with hypogonadism (Group 2). Men with hypogonadism were classified as either hypogonadotropic hypogonadism (Group 2a) or hypergonadotropic hypogonadism (Group 2b). Serum 25(OH)D levels were detected by liquid chromatography-tandem mass spectrometry in all cases. Morning total testosterone (TT), estradiol (E), dehydroepiandrosterone (DHEA), prolactin (PRL), sex hormone-binding globulin (SHBG), luteinizing hormone (LH), follicle-stimulating hormone (FSH), thyroid function, parathyroid and adrenal hormones, fasting blood glucose (FBG), fasting insulin (Fins) and hemoglobin A1c (HbA1c) were also assessed.

**Results:**

The prevalence of hypovitaminosis D in men with T2DM was up to 96.46%. Serum 25(OH)D levels were significantly lower in men with hypogonadism than those with eugonadism (16.65 ± 6.44 ng/mL *vs*. 18.17 ± 6.17 ng/mL, *P*=0.033). The lowest 25(OH)D level was observed in Group 2a (16.22 ± 6.52 ng/mL). After adjustment for the selected factors, serum 25(OH)D concentrations were shown to be positively correlated with TT concentrations (*r*=0.137, *P*=0.032). The relationship between 25(OH)D and testosterone was altered by age, duration of T2DM, body mass index, and HbA1c. Serum 25(OH)D level was positively associated with serum TT level in men with age <60 years (*r*=0.180, *P*=0.003), or with duration≥5 years (*r*=0.186, *P*=0.013), or with body mass index (BMI)≥28kg/m^2^ (*r*=0.431, *P*=0.000), or with HbA1c≥9% (*r*=0.145, *P*=0.031).

**Conclusions:**

These findings indicate that type 2 diabetes patients with hypogonadism have lower 25(OH)D levels than those without hypogonadism. There seems to be a positive association between the serum 25(OH)D and TT levels, which affected by age, duration, BMI, and HbA1c

## Introduction

Vitamin D deficiency has become a global public health concern (1–3). Over the past few decades, the prevalence of hypovitaminosis D has increased in the general population (4–6). Low vitamin D is associated with the risk of cardiovascular disorders, cancer, type 2 diabetes mellitus (T2DM), neurodegenerative diseases, hypogonadism and even life-threatening conditions (7).

Once vitamin D produced in the skin or ingested gets to the liver, it is mainly converted to 25-hydroxyvitamin D [25(OH)D] by the microsomal enzyme CYP2R1 with 25-hydroxylase activity, but also by CYP27A1 enzyme, widely distributed in several tissues with hydroxylase activity. 25(OH)D is the major circulating form of vitamin D that is measured to determine a person’s vitamin D status. However 25(OH)D is biologically inert and is transported to the kidneys where it is converted by the only enzyme with 1α hydroxylase activity, CYP27B1, to its fully active form 1,25-dihydroxyvitamin D [1,25(OH)2D] and most potent metabolite of vitamin D (8). Recent research highlights a close link between vitamin D deficiency and hypogonadism. A total of 4254 men in the Survey on Prevalence in East China for Metabolic Diseases and Risk Factors (SPECT-China) study showed that lower 25(OH)D levels were associated with lower total testosterone (TT) after multivariable adjustment (9). According to the findings of a meta-analysis, a low blood 25(OH)D level was slightly associated with a low testosterone level (10). Moreover, there has been some evidences indicating that the expression of the vitamin D receptor (VDR) and vitamin D metabolizing enzymes in the testis (11, 12). Notably, a clinical investigation discovered that type 2 diabetic patients with hypogonadism had a higher rate of vitamin D deficiency than those without hypogonadism (13).

It seems possible that vitamin D interacts with testosterone in patients with T2DM. However, no studies have investigated the prevalence of vitamin D deficiency in Chinese men with T2DM and hypogonadism. In addition, the relationship between 25(OH)D and testosterone still remains unknown. Hence, we evaluated serum 25(OH)D levels in diabetic men with and without hypogonadism and figured out the potential factors influencing the connection between vitamin D and testosterone.

## Materials and Methods

### Participants and Enrollment

From January 2015 to December 2016, we conducted a retrospective study among 367 males with T2DM from the Department of Endocrinology and Metabolic Diseases at Affiliated Hospital of Integrated Traditional Chinese and Western Medicine, Nanjing University of Chinese Medicine, including 254 with normal gonadal function (Group 1) and 113 with hypogonadism (Group 2).

Inclusion criteria were age >18 years, type 2 diabetes mellitus, and a previous screening for morning serum testosterone. Exclusion criteria included congenital hypogonadism, use of any agent that affected bone metabolism, malabsorption syndrome, testosterone supplementation, hypoparathyroidism, history of orchiectomy, and intake of fish oil. This research was approved by the institutional review board and the informed consent was obtained from all cases.

The definition of hypogonadism was based on standard criteria, particularly a morning (between 8 and 10 a.m.) serum testosterone concentration <11nmol/L with symptoms and signs of hypogonadal function (14). The differentiation between hypergonadotropic and hypogonadotropic hypogonadism was made according to serum luteinizing hormone (LH) levels, which would be elevated in the former, and low or normal in the latter. The LH threshold was 8.6 IU/L (the upper limit of the reference range in our hospital). Serum testosterone and LH levels were assessed in two different samples.

Although the deficiency and insufficiency cutoff values of vitamin D remain controversial, 25(OH)D concentrations were defined as insufficient (20-29.9ng/mL) or deficient (<20ng/mL) according to the Endocrine Society Clinical Practice Guideline (15). 25(OH)D is the primary metabolite of vitamin D and the best maker to monitor vitamin D status (16).

### Clinical Parameters

The medical data of all subjects were collected. Age, gender, height, weight, waist circumference, duration of T2DM and blood pressure were recorded, and body mass index (BMI) was also calculated.

### Biochemical Parameters

Serum 25(OH)D levels were detected by liquid chromatography-tandem mass spectrometry (LC-MS/MS). Fasting blood glucose (FBG) was measured by enzymatic test; glycated hemoglobin (HbA1c) analysis was performed with high performance liquid chromatography (HPLC); total cholesterol (TC), triglycerides (TG), low-density lipoprotein cholesterol (LDL-C), high-density lipoprotein cholesterol (HDL-C), albumin, calcium and phosphate were determined by enzymatic colorimetric test; serum creatinine was measured by enzymatic test. Levels of hormones, including fasting insulin (Fins), TT, sex hormone-binding globulin (SHBG), dehydroepiandrosterone (DHEA), LH, follicle-stimulating hormone (FSH), TT, estradiol (E2), prolactin (PRL), adrenocorticotropic hormone (ACTH), cortisol (F), thyroid-stimulating hormone (TSH), free thyroxine (fT4), free triiodothyronine (fT3), parathyroid hormone (PTH), were assayed by electrochemiluminescence immunoassay (ECLIA). Assays for urine albumin were also performed by immunity transmission turbidity. The levels of Homeostasis Model Assessment-insulin resistance (HOMA-IR), Homeostasis Model Assessment-β (HOMA-β), free testosterone, glomerular filtration rate (GFR) and urine albumin to creatinine ratio (ACR) were calculated. HOMA-IR was calculated according to the formula: Fins (uIU/L)×FBG (nmol/L)/22.5, and HOMA-β was calculated according to the formula: 20×Fins (uIU/L)/[FBG (nmol/L)-3.5]. In order to avoid the effects of seasonal variations, serum 25(OH)D levels were evaluated in plasma samples drawn from July through early September in 2015 and 2016.

### Statistical Analysis

Data analysis was performed through IBM SPSS Statistics, Version 21 (IBM Corporation, Armonk, NY, USA). Results were expressed as mean and standard deviation, median (interquartile range), or number (percentage). All the data passed the normality test except for those on FIns, HOMA-IR, HOMA-β, TG, ACR, LH, FSH and TSH and LH, which are expressed as median (interquartile range). Normal distribution was verified through the Kolmogorov-Smirnov test. Differences between two groups were evaluated by t-test and rank-sum test. Differences between frequencies were determined by chi-square test or Fisher’s exact test. Differences among groups were evaluated by one-way anova. Comparison among three groups was performed using Kruskal-Wallis H test. Correlations between 25(OH)D and age, BMI, HbA1c, FBG, FIns, HOMA-IR, HOMA-β, TT, free testosterone, sex hormone-binding globulin, dehydroepiandrosterone, luteinizing hormone, follicular stimulating hormone, estradiol, prolactin were evaluated by Pearson or Spearmen test. To address the independent relationships between 25(OH)D and the selected variable, a multiple linear regression model was used. A P-value of <0.05 was considered statistically significant.

## Results

**Figure 1:**
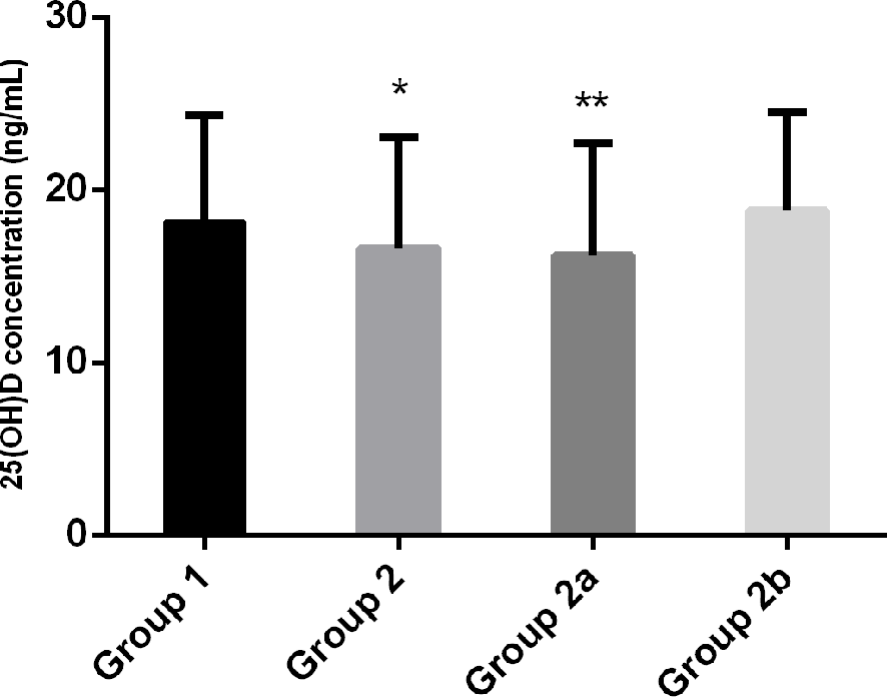
Vitamin D levels in patients with type 2 diabetes and normal testosterone levels (Group 1), patients with type 2 diabetes and hypogonadism (Group 2; hypogonadotropic hypogonadism [Group 2a] and hypergonadotropic hypogonadism [Group 2b]). Data are expressed as mean ± SD. *P < 0.05 *vs*. Group 1; **P < 0.05 *vs*. Group 1.

We investigated 367 men with T2DM (mean age 52.24 ± 11.51 years), including 254 men with normal gonadal function and 113 with hypogonadism. The main anthropometric, clinical, glucometabolic and hormonal characteristics are summarized in **Table 1**. The mean serum 25(OH)D level was 17.70 ± 6.29 ng/mL. The serum 25(OH)D level was significantly lower in men with T2DM with hypogonadism than in men with eugonadism (16.65 ± 6.44 ng/mL *vs*. 18.17 ± 6.17 ng/mL, *P*=0.033) (**Figure 1**). The prevalence of vitamin D deficiency (serum 25[OH]D<20 ng/mL) and insufficiency (serum 25[OH]D 20–29.9 ng/mL) was 62.99% and 33.47% in Group 1, and 69.03% and 27.43% in Group 2, respectively. No differences in vitamin D deficiency, insufficiency, or sufficiency prevalence have been found between Groups 1 and 2 (*P*=0.556).

**Table 1:** Clinical, metabolic, and hormonal parameters of patients.

	Group 1 n = 254	Group 2 n = 113	Group 2a n = 94	Group 2b n = 19	P
Age (yrs)	52.74 ± 11.26	51.12 ± 12.01	49.27 ± 11.46^▲^	60.32 ± 10.56^△,#^	<0.001
Duration (yrs)	4.98 ± 4.41	4.20 ± 3.50	4.33 ± 3.54	3.59 ± 3.36	0.176
Height (m^2^)	1.71 ± 0.06	1.71 ± 0.06	1.71 ± 0.06	1.71 ± 0.04	0.527
Weight (kg)	72.75 ± 10.79	77.36 ± 11.71*	77.95 ± 12.38^▲^	74.45 ± 7.15	0.001
BMI (kg/m^2^)	24.93 ± 3.22	26.29 ± 3.52*	26.46 ± 3.69^▲^	25.42 ± 2.38	0.001
25(OH)VD (ng/mL)	18.17 ± 6.17	16.65 ± 6.44*	16.22 ± 6.52^▲^	18.82 ± 5.71	0.026
<20, n (%)	160 (62.99%)	78 (69.03%)	67 (71.28%)	11 (57.90%)	
20-29.9, n (%)	85 (33.47%)	31 (27.43%)	24 (25.53%)	7 (36.84%)	
≥30, n (%)	9 (3.54%)	4 (3.54%)	3 (3.19%)	1 (5.26%)	
HbA1c (%)	9.74 ± 2.39	9.79 ± 2.23	9.78 ± 2.34	9.81 ± 1.58	0.986
FBG (mmol/L)	8.33 ± 3.08	9.14 ± 2.87*	9.01 ± 2.79	9.80 ± 3.26	0.035
FIns (uIU/ml)	6.24 (3.90-9.86)	8.96 (5.62-13.15)*	9.57 (5.82-14.00)^▲^	9.49 (5.79-12.85)^△^	<0.001
HOMA-IR	2.14 (1.35-3.59)	3.46 (2.13-5.51)*	3.72 (2.25-5.62)^▲^	3.40 (2.52-6.26)^△^	<0.001
HOMA-β	31.38 (16.34-63.21)	34.00 (19.64-66.31)	35.64 (20.88-66.45)	24.87 (15.90-82.24)	0.339
ALB (g/L)	42.66 ± 3.35	42.79 ± 3.80	42.73 ± 3.96	43.07 ± 3.00	0.882
TC (mmol/L)	4.55 ± 1.02	4.67 ± 1.23	4.72 ± 1.26	4.41 ± 1.05	0.337
TG (mmol/L)	1.34 (0.90-1.87)	2.1 (1.23-3.84)*	2.22 (1.34-4.37)	1.41 (1.01-2.75)	<0.001
LDL (mmol/L)	3.08 ± 0.91	2.86 ± 1.02*	2.88 ± 1.03	2.76 ± 1.04	0.114
HDL (mmol/L)	1.12 ± 0.34	0.93 ± 0.27*	0.91 ± 0.26^▲^	1.01 ± 0.29	<0.001
Cr (umol/L)	73.34 ± 19.38	70.05 ± 14.81	69.32 ± 14.21	73.68 ± 17.44	0.176
GFR (ml/min·1.73m^2^)	111.85 ± 33.39	126.29 ± 40.93*	130.86 ± 41.62^▲^	103.67 ± 28.74^#^	<0.001
Ca (mmol/L)	2.25 ± 0.11	2.24 ± 0.11	2.24 ± 0.11	2.25 ± 0.11	0.863
P (mmol/L)	1.18 ± 0.20	1.15 ± 0.19	1.14 ± 0.20	1.18 ± 0.13	0.277
PTH (pg/ml)	41.88 ± 14.25	41.10 ± 16.73	41.62 ± 17.30	38.54 ± 13.70	0.649
ACR (mg/g)	6.66 (3.81-15.94)	11.03 (4.65-41.99)*	11.39 (4.80-42.21)^▲^	11.60 (3.69-62.17)	0.004
TT (nmol/L)	16.53 ± 4.20	8.65 ± 1.71*	8.56 ± 1.75^▲^	9.12 ± 1.44^△^	<0.001
fT (pmol/L)	333.18 ± 73.64	222.06 ± 60.61*	223.91 ± 61.42^▲^	212.89 ± 57.10^△^	<0.001
SHBG (nmol/L)	36.79 ± 16.90	20.75 ± 9.89*	19.73 ± 8.97^▲^	25.76 ± 12.67^△^	<0.001
DHEA (umol/L)	5.55 ± 2.44	6.27 ± 2.57*	6.43 ± 2.53^▲^	5.47 ± 2.68^#^	0.011
LH (mIU/mL)	6.18 (4.55-8.53)	5.47 (3.78-7.34)*	4.76 (3.52-6.58)^▲^	10.42 (9.34-13.00)^△,#^	<0.001
FSH (mIU/mL)	7.01 (4.89-10.25)	5.84 (3.65-9.64)*	5.12 (3.42-7.56)^▲^	12.45 (9.74-17.53)^△, #^	<0.001
E2 (pmol/L)	104.20 ± 43.09	83.51 ± 39.13*	84.13 ± 39.66^▲^	80.43 ± 37.22^△^	<0.001
PRL (uIU/ml)	292.91 ± 158.36	288.05 ± 138.11	284.57 ± 136.81	306.22 ± 147.46	0.826
F (nmol/L)	470.91 ± 158.19	482.85 ± 177.96	479.46 ± 178.06	499.23 ± 181.38	0.731
ACTH (pg/ml)	34.96 ± 19.15	34.93 ± 24.96	33.50 ± 25.29	41.93 ± 22.60	0.284
TSH (uIU/ml)	1.61 (1.09-2.29)	1.55 (1.13-2.27)	1.48 (1.13-2.27)	1.74 (1.05-2.31)	0.843
fT3 (pmol/L)	4.96 ± 0.72	4.88 ± 0.64	4.87 ± 0.66	4.95 ± 0.51	0.526
fT4 (pmol/L)	17.05 ± 2.95	17.18 ± 2.24	17.23 ± 2.32	16.94 ± 1.82	0.836

Data are expressed as mean ± SD, median (interquartile range), number (percentage).

Group 1, patients with type 2 diabetes and normal gonadal function; Group 2, patients with type 2 diabetes and hypogonadism; Group 2a, patients with type 2 diabetes and hypogonadotropic hypogonadism; Group 2b, patients with type 2 diabetes and hypergonadotropic hypogonadism.

P value represents statistically significant difference between Group 1, Group 2a and Group 2b (ANOVA).

*P < 0.05, Group 1 vs. Group 2; ^▲^P < 0.05, Group 1 vs. Group 2a; ^△^P < 0.05, Group 1 vs. Group 2b; ^#^P < 0.05, Group 2a vs. Group 2b (t-test or rank-sum test).

25(OH)VD, 25-hydroxyvitamin D; ACR, urine albumin to creatinine ratio; ACTH, adrenocorticotropic hormone; ALB, albumin; BMI, body mass index; Ca, calcium; Cr, creatinie; DHEA, dehydroepiandrosterone; E2, estradiol; F, cortisol; FBG, fasting blood glucose; Fins, fasting insulin; FSH, follicular stimulating hormone; fT, free testosterone; fT3, free triiodothyronine; fT4, free thyroxine; GFR, glomerular filtration rate; HbA1c, hemoglobin A1c; HDL, high-density lipoprotein; LDL, low-density lipoprotein; LH, luteinizing hormone; P, phosphorus; PRL, prolactin; PTH, parathyroid hormone; SHBG, sex hormone-binding globulin; TC, total cholesterol; TG, triglyceride; TSH, thyroid-stimulating hormone; TT, total testosterone.

Group 2 was further categorized into Group 2a (mean age 49.27 ± 11.46 years, BMI 26.46 ± 3.69 kg/m^2^), which included 94 men with hypogonadotropic hypogonadism (low testosterone+low or normal LH) and Group 2b (mean age 60.32 ± 10.56 years, BMI 25.42 ± 2.38 kg/m^2^), which included 19 men with hypergonadotropic hypogonadism (low testosterone+high LH) (**Table 1**). For serum 25(OH)D levels, we found a statistically significant difference between Groups 1 and 2a (18.17 ± 6.17 ng/mL *vs*. 16.22 ± 6.52 ng/mL, *P*=0.011), but not between Groups 1 and 2b (18.17 ± 6.17 ng/mL *vs*. 18.82 ± 5.71 ng/mL, *P*=0.659) (**Figure 1**). Besides, there was no significant difference in serum 25(OH)D levels between Groups 2a and 2b (*P*=0.098). No differences in vitamin D deficiency, insufficiency, or sufficiency prevalence have been found among Groups 1, 2a, and 2b (*P*=0.301) (**Table 1**).

Serum 25(OH)D showed significant differences between Groups 1, 2a, and 2b (*P*=0.025) in men with serum 25(OH)D levels <20 ng/mL (14.43 ± 3.82 ng/mL *vs*. 13.26 ± 4.14 ng/mL *vs*. 12.96 ± 4.20 ng/mL), but not in men with serum 25(OH)D of 20–29.9 ng/mL (*P*=0.701) (**Table 2**).

**Table 2:** Serum 25(OH)D levels of patients in Group 1, Group 2, Group 2a and Group 2b.

	Group 1 n = 254	Group 2 n = 113	Group 2a n = 94	Group 2b n = 19	P
<20	14.43 ± 3.82	13.26 ± 4.14*	12.96 ± 4.20^▲^	15.08 ± 3.32	0.025
20-29.9	23.67 ± 2.74	23.28 ± 2.53	23.40 ± 2.53	22.86 ± 2.69	0.701
≥30	32.49 ± 2.29	31.45 ± 1.20	31.37 ± 1.46	31.7	NA

Data are expressed as mean ± SD, median (interquartile range).

Group 1, patients with type 2 diabetes and normal testosterone levels; Group 2, patients with type 2 diabetes and hypogonadism; Group 2a, patients with type 2 diabetes and hypogonadotropic hypogonadism; Group 2b, patients with type 2 diabetes and hypergonadotropic hypogonadism.

P value represents statistically significant difference between Group 1, Group 2a and Group 2b (ANOVA).

*P < 0.05, Group 1 vs. Group 2; ^▲^P < 0.05, Group 1 vs. Group 2a (t-test or rank-sum test).

NA, not applicable.

Our correlation analysis showed a significant association between serum vitamin D concentration and age (*r*=0.150, *P*=0.004), duration (*r*=-0.124, *P*=0.017), BMI (*r*=-0.172, *P*=0.001), FBG (*r*=-0.108, *P*=0.039), Fins (*r*=-0.131, *P*=0.012), HOMA-IR (*r*=-0.157, *P*=0.003), TT (*r*=0.142, *P*=0.006), and SHBG levels (*r*=0.187, *P*=0.000) (**Table 3**). Multiple linear regression analysis was performed to investigate the association between serum 25(OH)D level and testosterone levels (**Table 4**). In Model 1, the level of serum 25(OH)D was significantly associated with that of TT without adjustment for any factors (*r* =0.174, *P*=0.006). In Model 2, serum 25(OH)D was significantly associated with the TT after adjustment for age, duration, BMI (*r*=0.135, *P*=0.035). In Model 3, serum 25(OH)D was significantly associated with the TT after adjustment for HbA1c, FBG, FIns, HOMA-IR, HOMA-β (*r*=0.174, *P*=0.006). In Model 4, serum 25(OH)D was significantly associated with the TT after adjustment for LH, FSH, E_2_, DHEA, PRL (*r*=0.177, *P*=0.005). In Model 5, serum 25(OH)D was significantly associated with the TT after adjustment for all the factors above (*r*=0.137, *P*=0.032).

**Table 3:** Relationship between serum 25(OH)D and age, BMI, sex hormone and glycometabolic parameters.

	Age	Duration	BMI	HbA1c	FBG	FIns	HOMA-IR	HOMA–β
r	0.150	-0.124	-0.172	-0.035	-0.108	-0.131	-0.157	-0.008
P	0.004	0.017	0.001	0.503	0.039	0.012	0.003	0.878
	T	fT	SHBG	DHEA	LH	FSH	E2	PRL
r	0.142	0.011	0.187	-0.034	0.091	0.101	-0.042	-0.074
P	0.006	0.838	0.000	0.520	0.080	0.052	0.419	0.159

r, correlation coefficient.

BMI, body mass index; DHEA, dehydroepiandrosterone; E2, estradiol; FBG, fasting blood glucose; Fins, fasting insulin; FSH, follicular stimulating hormone; fT, free testosterone; HbA1c, hemoglobin A1c; LH, luteinizing hormone; PRL, prolactin; SHBG, sex hormone-binding globulin; TT, total testosterone.

**Table 4:** Multiple linear regression analysis of 25(OH)D, testosterone and other parameters.

Dependent variable	Slope	Model 1	Model 2	Model 3	Model 4	Model 5
VD	Beta	0.174	0.135	0.174	0.177	0.137
	P	0.006	0.035	0.006	0.005	0.032

Model 1: Not adjusted; Model 2: Adjusted age, duration, BMI; Model 3: Adjusted HbA1c, FBG, FIns, HOMA-IR, HOMA-β; Model 4: Adjusted LH, FSH, E2, DHEA, PRL; Model 5: Adjusted age, duration, BMI, HbA1c, FBG, FIns, HOMA-IR, HOMA-β, LH, FSH, E2, DHEA, PRL.

**Figure 2:**
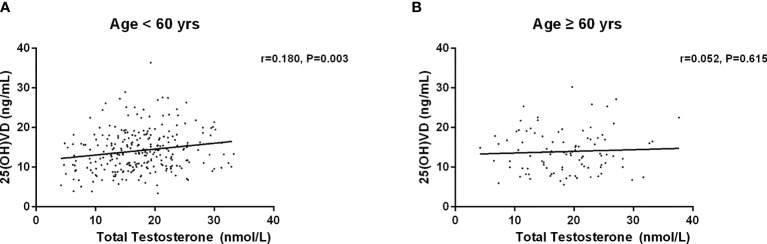
Relationship between serum 25(OH)D and total testosterone in patients less than 60 years old (n=271) and more than 60 years old (n=96). **(A)** Patients with age < 60 years old; **(B)** Patients with age ≥ 60 years old.

**Figure 3:**
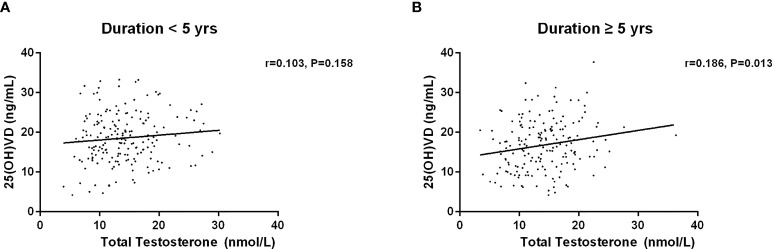
Relationship between serum 25(OH)D and total testosterone in patients with duration less than 5 years (n=189) and more than 5 years (n=178). **(A)** Patients with duration < 5 years; **(B)** Patients with duration ≥ 5 years.

**Figure 4:**
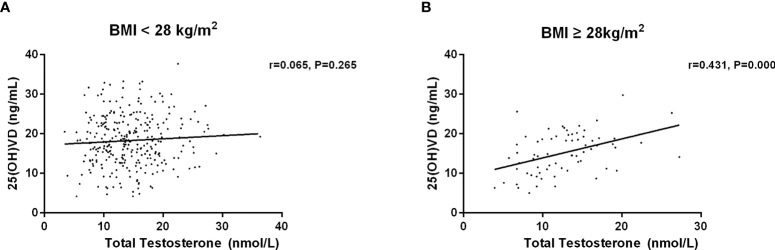
Relationship between serum 25(OH)D and total testosterone in patients with BMI less than 28kg/m^2^ (n=298) and more than 28kg/m^2^ (n=69). **(A)** Patients with BMI < 28 kg/m^2^; **(B)** Patients with BMI ≥ 28kg/m^2^.

**Figure 5:**
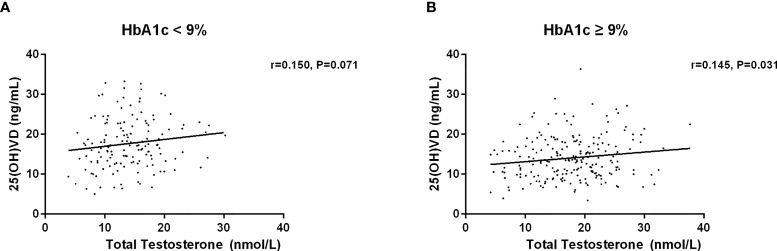
Relationship between serum 25(OH)D and total testosterone in patients with HbA1c less than 9% (n=146) and more than 9% (n=221). **(A)** Patients with HbA1c < 9%; **(B)** Patients with HbA1c ≥ 9%.

Then, we investigated the association between serum 25(OH)D and testosterone levels after age, duration, BMI and HbA1c stratification (**Tables 5**–**8**). First, we observed that serum 25(OH)D level was positively associated with TT level in younger men (age <60 years) (*r*=0.180, *P*=0.003), but not in men aged ≥60 years (*r*=0.052, *P*=0.615) (**Figure 2**). Second, serum 25(OH)D level was significantly related to TT level in cases with duration≥5 years (*r*=0.186, *P*=0.013), but not in cases with duration<5 years (*r*=0.103, *P*=0.158) (**Figure 3**). Third, serum 25(OH)D level was significantly related to TT level in men with BMI≥28kg/m^2^ (*r*=0.431, *P*=0.000), but not in men with BMI<18.5kg/m^2^ (*r*=0.065, *P*=0.265) (**Figure 4**). At last, serum 25(OH)D level was significantly associated with TT level in cases with HbA1c≥9% (*r*=0.145, *P*=0.031), but not in cases with HbA1c<9% (r=0.150, P=0.071) (**Figure 5**). Hence, age, duration, BMI and HbA1c affected the association between 25(OH)D and testosterone.

**Table 5:** Relationship between serum 25(OH)D and total testosterone after age stratification.

Age	r	P
20-29 yrs (n=11)	0.323	0.333
30-39 yrs (n=42)	0.209	0.184
40-49 yrs (n=75)	0.130	0.267
50-59 yrs (n=143)	0.149	0.076
60-69 yrs (n=73)	0.071	0.552
>70 yrs (n=23)	-0.031	0.887

r, correlation coefficient.

**Table 6:** Relationship between serum 25(OH)D and total testosterone after duration stratification.

Duration (years)	r	P
<1	0.213	0.427
1-5	0.064	0.401
5-10	0.165	0.079
≥10	0.265	0.036

r, correlation coefficient.

**Table 7:** Relationship between serum 25(OH)D and total testosterone after BMI stratification.

BMI (kg/m^2^)	r	P
<18.5	0.100	0.873
18.5-24	0.039	0.667
24-28	0.092	0.236
≥28	0.431	0.000

**Table 8:** Relationship between serum 25(OH)D and total testosterone after HbA1c stratification.

HbA1c (%)	r	P
<7.5	0.092	0.472
7.5-9	0.176	0.113
≥9	0.145	0.031

## Discussion

Hypovitaminosis D is a major public health concern around the world (5, 17–19). In individuals with type 2 diabetes, the prevalence of hypovitaminosis D has grown. A large-scale prospective study has reported that approximately 50% of the patients with type 2 diabetes showed low vitamin D concentrations (<50 nmol/L [20 ng/mL]) (20). Additionally, a cross-sectional survey of 280 patients with diabetes demonstrated that 34.2% of the patients showed low vitamin D levels (21). In our study, > 90% of men with T2DM showed vitamin D deficiency or insufficiency. Our results concur with those of a previous study that observed a hypovitaminosis D rate as high as 97.8% in Chinese patients with T2DM (22). Another study performed in Shanghai, including 861 patients with T2DM, reported that approximately 90% of the patients had low vitamin D concentrations (23). However, the serum 25(OH)D cutoff value used by studies tends to vary; a high value may result in an inaccurately estimated high prevalence of hypovitaminosis D, leading to aggressive but unnecessary vitamin D supplementation. According to a recent survey in China, the optimal cutoff concentration was 22 ng/mL (55 nmol/L) (24). As previously stated, we defined insufficiency as serum 25(OH)D of 12–20 ng/mL and deficiency as serum 25(OH)D levels of <12 ng/mL in our region.

To our knowledge, previous studies have described the vitamin D profile in patients with concomitant diabetes and hypogonadism. Recent studies reported that patients with T2DM and hypogonadism, particularly those with hypogonadotropic hypogonadism, showed the lowest serum 25(OH)D levels and the highest prevalence of vitamin D deficiency (13). Remarkably, our study results also showed that patients with T2DM and hypogonadotropic hypogonadism had the lowest serum 25(OH)D concentrations. A few studies reported that hypovitaminosis D was associated with low serum testosterone, which may usually be accompanied by low gonadotropin (25–27). A cross-sectional survey of 3369 elder men aged 40-79 years revealed that vitamin D deficiency was significantly associated with compensated and secondary hypogonadism (25). Given the relationship between 25(OH)D and hypogonadism, including testosterone and LH, it is plausible that low serum vitamin D levels have an effect on multiple levels of the HPT axis. Additionally, these results implied that low vitamin D and hypogonadism are both indicators of aging, possibly sharing common underlying etiologies. Besides, secondary hypogonadism is predominately associated with overweight and obesity, which makes it likely that increased aromatization of T to E2 in adipocytes, increased insulin resistance, and proinflammatory cytokine production (tumor necrosis factor a and interleukin 6) from adipose tissues may have a negative impact on the vitamin D endocrine axis (28). Hence, we hypothesized that VDR might interfere with gonadotropin synthesis. It is known that VDRs are abundant in the adenohypophysis and LH is produced exclusively by the gonadotropic cells of the anterior pituitary (29). It is reasonable to conclude that VDRs may participate in LH production and that efficient VDR C-alleles may directly regulate the transcriptional activity of genes associated with LH synthesis. Conversely, the finding of decreased vitamin D levels in hypogonadotropic hypogonadism patients compared to hypergonadotropic hypogonadism patients contradicts previous research demonstrating that the testis is an important site of vitamin D hydroxylation (30, 31). In addition, one study discovered high levels of CYP2R1 expression in Leydig cells, implying a link between vitamin D metabolism and testis function (11). Further investigations are needed to elucidate these issues.

Furthermore, there was no significant difference in blood 25(OH)D concentrations between Groups 1 and 2, and this concentration had no relationship with vitamin D deficiency, insufficiency, or sufficiency. In contrast, recent research has shown various relationships between 25(OH) D levels and vitamin D deficiency, insufficiency, or sufficiency in men with T2DM and those without hypogonadism (13). The prevalence of vitamin D deficiency and insufficiency in men with T2DM concomitant with hypogonadism was 50.9% and 33.3%, respectively, in contrast to 18.3% and 60.5% in men with T2DM with eugonadal function (13). It is well known that vitamin D status is affected by several factors, including geographical latitude, season, ethnicity, age, sex, genetics, and renal function (17). In our study, ethnicity and geography may account for the differences in 25(OH)D arising from vitamin D deficiency, insufficiency, or sufficiency.

Our finding indicated a positive association between serum 25(OH)D and TT levels, which concurs with the findings of previous studies (25, 27, 32–34). Multiple linear regression analysis showed that serum 25(OH)D was significantly associated with total serum testosterone, age, BMI, FBG, FIns, HOMA-IR, and SHBG. It is worth noting that age, duration, BMI and HbA1c affected the connection between 25(OH)D and testosterone. In contrast to previous studies (35–38), our results revealed that no significant correlation between serum 25(OH)D and testosterone was identified in younger males. A recent study that investigated 122 men with diabetes (mean age 55 ± 8.85 years) revealed an insignificant association between serum 25(OH)D and testosterone (13). Interestingly, we found that serum 25(OH)D was positively associated with total serum testosterone in younger men (age <60 years), but not in men aged ≥60 years. Recent similar results reported that 25(OH)D level was positively correlated with TT level in males aged 18-60 years (39). However, another indicated a positive association between 25 (OH) D level and free testosterone level only in men aged more than 70 years, but not in men aged below 70 years (26). It’s worth noting that the age of the participants between 40-75 years old in the latter study were much older. Apart from age, duration, BMI and HbA1c were observed to affect the association between serum 25(OH)D and testosterone in men with T2DM.

Following are the limitations of this study: (a) Our results were based on cross-sectional data, and it was hard to unveil causal relationships among variables. (b) Smoking status, sexual symptoms and VDR polymorphism were not investigated in this study. (c) Our results may not be generalizable across all populations owing to the limited numbers and ethnicities of patients analyzed in this study. Further investigations are needed to warrant the causal association between hypovitaminosis D and multilevel disorders of the hypothalamic-pituitary-testicular (HPT) axis.

## Conclusion

This study verifies the high prevalence of low vitamin D level in patients with T2DM. Notably, deficient serum 25(OH)D was mainly observed in men with T2DM with hypogonadotropic hypotestosteronemia, which indicates a positive association between the serum 25(OH)D and total serum testosterone concentrations after adjustment for age, duration, BMI, HbA1c, FBG, FIns, HOMA-IR, HOMA-β, LH, FSH, E2, DHEA, PRL. Additionally, age, duration, BMI and HbA1c affected the association between serum 25(OH)D and testosterone. Further investigations are needed to warrant the causal association between serum vitamin D and the action of the HPT axis and uncover the underlying mechanisms.

## Data Availability Statement

The raw data supporting the conclusions of this article will be made available by the authors, without undue reservation.

## Ethics Statement

Written informed consent was obtained from the individual(s) for the publication of any potentially identifiable images or data included in this article.

## Author Contributions

XHu, XHa, YC, TG, GC, and CL conceived and designed research. XHu, XHa, PX, XW, TG, ZH, YS, GC, and CL collected data and conducted research. TG, ZH, GC, and CL analyzed and interpreted data. XHu wrote the initial paper. YC, GC, and CL revised the paper. CL had primary responsibility for final content. All authors contributed to the article and approved the submitted version.

## Funding

This study was funded by grants from National Natural Science Foundation of China (Grant no. 81800756 to GC).

## Conflict of Interest

The authors declare that the research was conducted in the absence of any commercial or financial relationships that could be construed as a potential conflict of interest.

## Publisher’s Note

All claims expressed in this article are solely those of the authors and do not necessarily represent those of their affiliated organizations, or those of the publisher, the editors and the reviewers. Any product that may be evaluated in this article, or claim that may be made by its manufacturer, is not guaranteed or endorsed by the publisher.
